# Neonatal-onset of multiple minute digitate hyperkeratosis with photosensitivity and linear epidermal nevus: A case report

**DOI:** 10.1016/j.jdcr.2026.04.033

**Published:** 2026-04-23

**Authors:** Nermeen Elhofy, Shada Alanazi, Sara Almukhaimar, Hadeel Almajid, Ali Aldandan

**Affiliations:** a Department of Dermatology, Prince Saud Bin Jalawy Hospital, Ministry of Health, Al-Ahsa, Saudi Arabia; b College of Medicine, King Faisal University, Al-Ahsa, Saudi Arabia; c Department of Dermatology, Almoosa Specialist Hospital, Almoosa Health Group, Al-Ahsa, Saudi Arabia; d College of Medicine, Al Faisal University, Riyadh, Saudi Arabia; e Department of Pathology, Prince Saud Bin Jalawy Hospital, Ministry of Health, Al-Ahsa, Saudi Arabia

**Keywords:** birth-onset hyperkeratosis, keratinization disorders, linear epidermal nevus, multiple minute digitate hyperkeratosis, pediatric dermatology, photosensitivity

## Introduction

Multiple minute digitate hyperkeratosis (MMDH), first described by Goldstein in 1967, is a rare dermatological condition characterized by numerous asymptomatic, small, nonfollicular, digitiform keratotic projections. The lesions predominantly affect the trunk and extremities.^1^

We report an atypical case of MMDH in a 14-y-old male with lesions present since birth, associated with subjective photosensitivity and a congenital linear epidermal nevus. This presentation expands the phenotypic spectrum of MMDH and provides insight into its pediatric course.

## Case report

A 14-y-old male presented with progressively worsening generalized dry skin characterized by finger-like projections and flattened keratotic papules affecting the face, trunk, and extremities since birth.

Cutaneous findings were first noted at birth as perinasal minute spiky keratinous projections following an uncomplicated full-term delivery. Within the first weeks of life, the patient developed facial erythema and numerous minute, nonfollicular, yellowish-white keratinous spicules, accompanied by subjective photosensitivity, with facial erythema reported after sun exposure. The condition progressed throughout childhood and adolescence, resulting in generalized involvement. There was no family history of similar dermatological conditions, and the parents were nonconsanguineous. Past medical history was notable only for well-controlled bronchial asthma.

**Fig 1:**
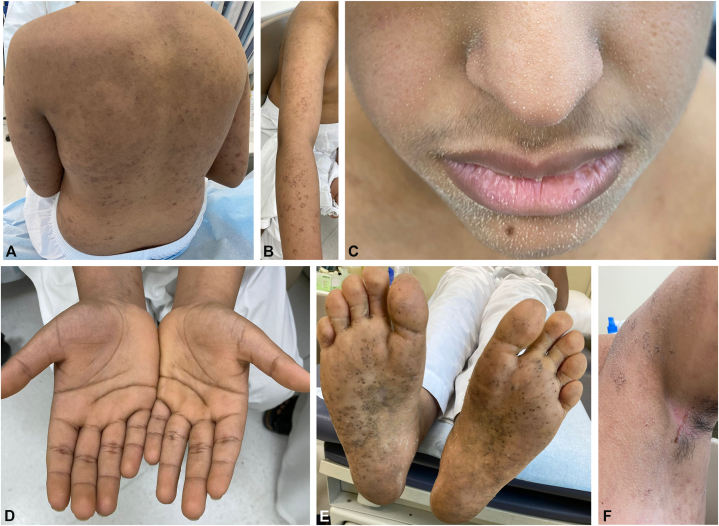
Clinical features of multiple minute digitate hyperkeratosis in a 14-y-old male. **(A**, **B)** Nonfollicular brown flattened-to-dome-shaped keratotic papules (2-3 mm) on extremities and trunk. **(C)** finger-like white hyperkeratotic projections on the face. **(D)** Mild palmar keratoderma. **(E)** Plantar keratoderma with pitted keratolysis showing crateriform pits. **(F)** Linear epidermal nevus presenting as a unilateral hyperkeratotic verrucous plaque on the left axilla.

Cutaneous examination revealed multiple flattened to dome-shaped brownish keratotic papules distributed over the trunk and extremities (Fig 1, *A* and *B*), as well as numerous nonfollicular white filiform hyperkeratotic projections on the face measuring approximately 1–2 mm in diameter (Fig 1, *C*). Additional findings included palmar keratoderma (Fig 1, *D*), plantar keratoderma with pitted keratolysis (Fig 1, *E*), and a congenital linear epidermal nevus involving the left axilla (Fig 1, *F*). Hair, nails, oral mucosa, and dentition were unremarkable.

**Fig 2:**
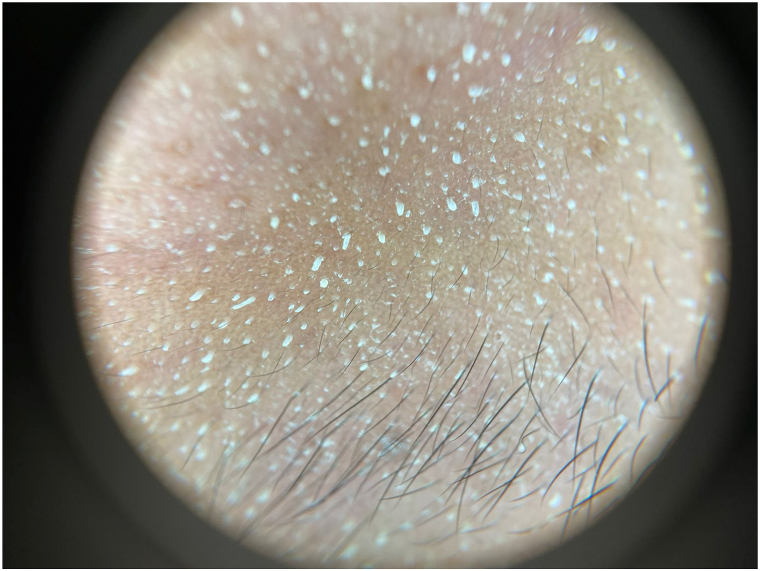
Dermoscopic features of multiple minute digitate hyperkeratosis. Multiple filamentous white keratotic projections appear as “spikes” with nonfolliculocentric distribution against a homogeneous background without vascular structures or pigment network.

Dermoscopic examination demonstrated multiple white filamentous keratotic projections with a nonfolliculocentric distribution on a homogeneous background, without associated vascular structures or pigment network (Fig 2).

**Fig 3:**
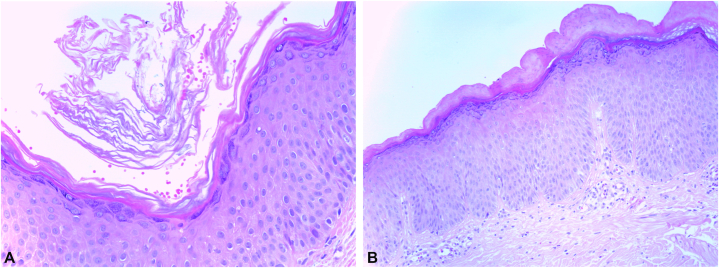
Histopathologic findings of multiple minute digitate hyperkeratosis from right upper arm biopsy. **(A)** Columns of orthokeratotic hyperkeratosis overlying invaginated epidermis without edema, inflammation, or dyskeratotic keratinocytes (H&E; original magnification, ×200). **(B)** Hyperkeratosis with focal parakeratosis and mild epidermal tenting (H&E; original magnification, ×200).

Histopathologic examination of a punch biopsy obtained from a right upper arm lesion revealed features consistent with multiple minute digitate hyperkeratosis. Findings included columns of orthokeratotic hyperkeratosis overlying invaginated epidermis without associated edema, inflammation, or dyskeratotic keratinocytes (Fig 3, *A*), along with hyperkeratosis showing focal parakeratosis and mild epidermal tenting (Fig 3, *B*). No biopsy was obtained from the axillary linear epidermal nevus due to its characteristic clinical appearance.

Laboratory evaluation revealed isolated vitamin D deficiency. Whole exome sequencing did not identify pathogenic variants.

The differential diagnosis included keratosis pilaris variants (spinulosa and atrophicans), congenital ichthyosis, follicular hyperkeratotic spicules, erythrokeratoderma, atrophoderma vermiculatum, and lichen spinulosus. The diagnosis of multiple minute digitate hyperkeratosis was established based on clinicopathologic correlation.

Initial treatment consisted of oral isotretinoin (0.25 mg/kg/d) in combination with sunscreen and vitamin D_3_ supplementation (cholecalciferol 50,000 IU weekly). Pitted keratolysis was treated with a topical clindamycin 1% solution. Isotretinoin was discontinued due to palpitations, and therapy was transitioned to topical keratolytic agents, including 10% urea cream, 5% salicylic acid, and 0.05% tretinoin cream. The patient was lost to follow-up for 2 y and returned with a similar clinical presentation without significant progression. Given increased age and body weight, oral isotretinoin was reinitiated at 0.5 mg/kg/d in combination with topical keratolytics, resulting in marked improvement of hyperkeratotic and spiky lesions within 1 mo, without adverse effects.

## Discussion

MMDH belongs to the broader clinicopathological spectrum of digitate keratoses, a heterogeneous group of inherited and acquired disorders characterized by spiny or filiform keratotic projections arising from the follicular infundibulum. Conditions within this group include MMDH, lichen spinulosus, and spiny keratoderma, as well as several acquired entities. These include phrynoderma associated with nutritional deficiencies; arsenical keratosis resulting from chronic arsenic exposure; multiple filiform verrucae caused by human papillomavirus infection; post-irradiation digitate keratosis; trichodysplasia spinulosa related to polyomavirus infection; and hyperkeratotic spicules, which have been linked to systemic disorders such as multiple myeloma.^1^^,^^2^ Accurate differentiation among these entities relies on clinicopathological correlation, assessment of systemic associations and exposure history, and—when applicable—molecular findings.

MMDH has been classified into 3 patterns: autosomal dominant familial forms with early onset, sporadic acquired cases, and post-inflammatory variants.^1^^,^^3^ Postinflammatory MMDH has been reported following inflammatory dermatoses, topical therapies, ultraviolet exposure, and radiation.^4^ Diagnosis is established through clinicopathological correlation. Histopathological features classically include columns of orthokeratotic hyperkeratosis arising from a tented or mildly acanthotic epidermis, with sparing of the intervening epidermis. Post-inflammatory variants may additionally demonstrate epidermal invagination and focal parakeratosis.^1^ The histopathological findings in the present case were consistent with these established criteria.

MMDH has been documented across a wide age range. In the largest available review of 73 cases, onset ranged from 3 mo to 81 y (mean, 48.8 y).^1^ The present case is notable for congenital onset with persistence into adolescence; however, clinical presentation occurred at 14 y. The absence of a family history distinguishes this case from inherited early-onset forms^5^^,^^6^ and supports classification as sporadic congenital MMDH. Although MMDH most commonly involves the trunk and extremities, a recognized distribution—facial involvement has been reported only rarely.^7^^,^^8^ The perinasal onset and prominent facial involvement observed here are distinctly uncommon and, in conjunction with the congenital course, serve to expand the recognized anatomic spectrum of the condition.

Two associated findings warrant consideration. The patient reported photosensitivity with facial erythema on sun exposure, a feature not described in the 73-case review,^1^ suggesting that it may be underrecognized or incidental; however, the absence of formal photobiologic testing precludes definitive conclusions. The coexistence of a congenital linear epidermal nevus is also unusual. Epidermal nevi arise from postzygotic somatic mosaicism involving FGFR3, PIK3CA, or RAS pathways,^9^ which are genetically distinct from disorders of keratinization; moreover, this association has not been previously reported in MMDH, supporting a coincidental rather than pathogenetically linked relationship.

Management remains empirical, with no established standard of care.^3^^,^^10^ Topical keratolytics, emollients, and retinoids have demonstrated variable efficacy. In this case, systemic isotretinoin administered at a higher weight-adjusted dose resulted in marked improvement, supporting its potential utility in extensive disease. Recurrence following treatment interruption underscores the need for individualized long-term management strategies.

In conclusion, this case expands the clinical spectrum of MMDH by documenting sporadic congenital onset with persistence into adolescence, facial involvement, subjective photosensitivity, and concurrent linear epidermal nevus.

## Conflict of interest

None disclosed.
